# Sentinel lymph node biopsy *versus* axillary lymph node dissection in breast cancer patients undergoing mastectomy with one to two metastatic sentinel lymph nodes: sub-analysis of the SINODAR-ONE multicentre randomized clinical trial and reopening of enrolment

**DOI:** 10.1093/bjs/znad215

**Published:** 2023-07-20

**Authors:** Corrado Tinterri, Giuseppe Canavese, Wolfgang Gatzemeier, Erika Barbieri, Alberto Bottini, Andrea Sagona, Giulia Caraceni, Alberto Testori, Simone Di Maria Grimaldi, Carla Dani, Luca Boni, Paolo Bruzzi, Bethania Fernandes, Marta Scorsetti, Alberto Zambelli, Damiano Gentile, SINODAR-ONE Collaborative Group, Massimo Maria Grassi, Olindo Custodero, Vito Leopoldo Troilo, Mario Taffurelli, Maria Cristina Cucchi, Valentina Galluzzo, Carlo Cabula, Roberta Cabula, Maria Grazia Lazzaretti, Francesco Caruso, Gaetano Castiglione, Simona Grossi, Maria Saveria Tavoletta, Camilla Rossi, Annalisa Curcio, Daniele Friedman, Piero Fregatti, Carla Magni, Giovanni Tazzioli, Simona Papi, Riccardo Giovanazzi, Camelia Chifu, Rossella Bettini, Modestino Pezzella, Silvia Michieletto, Tania Saibene, Manuela Roncella, Matteo Ghilli, Andrea Sibilio, Anna Cariello, Saverio Coiro, Giuseppe Falco, Emanuele Zarba Meli, Lucio Fortunato, Luigi Ciuffreda, Roberto Murgo, Claudio Battaglia, Luca Rubino, Nicoletta Biglia, Valentina Bounous, Francesca Angela Rovera, Corrado Chiappa, Giovanni Pollini, Sara Mirandola, Graziano Meneghini, Francesco Di Bartolo, Oreste Davide Gentilini

**Affiliations:** Breast Unit, IRCCS Humanitas Research Hospital, Milan, Italy; Department of Biomedical Sciences, Humanitas University, Milan, Italy; Breast Unit, IRCCS Humanitas Research Hospital, Milan, Italy; Breast Unit, IRCCS Humanitas Research Hospital, Milan, Italy; Breast Unit, IRCCS Humanitas Research Hospital, Milan, Italy; Breast Unit, IRCCS Humanitas Research Hospital, Milan, Italy; Breast Unit, IRCCS Humanitas Research Hospital, Milan, Italy; Breast Unit, IRCCS Humanitas Research Hospital, Milan, Italy; Breast Unit, IRCCS Humanitas Research Hospital, Milan, Italy; Breast Unit, IRCCS Humanitas Research Hospital, Milan, Italy; Department of Epidemiology, Biostatistics and Clinical Trials, IRCCS S. Martino, IST, Genoa, Italy; Department of Epidemiology, Biostatistics and Clinical Trials, IRCCS S. Martino, IST, Genoa, Italy; Department of Epidemiology, Biostatistics and Clinical Trials, IRCCS S. Martino, IST, Genoa, Italy; Department of Pathology, IRCCS Humanitas Research Hospital, Milan, Italy; Department of Biomedical Sciences, Humanitas University, Milan, Italy; Department of Radiotherapy and Radiosurgery, IRCCS Humanitas Research Hospital, Milan, Italy; Department of Biomedical Sciences, Humanitas University, Milan, Italy; Medical Oncology and Haematology Unit, IRCCS Humanitas Research Hospital, Milan, Italy; Breast Unit, IRCCS Humanitas Research Hospital, Milan, Italy; Department of Biomedical Sciences, Humanitas University, Milan, Italy

## Abstract

**Background:**

The initial results of the SINODAR-ONE randomized clinical trial reported that patients with T1–2 breast cancer and one to two macrometastatic sentinel lymph nodes treated with breast-conserving surgery, sentinel lymph node biopsy only, and adjuvant therapy did not present worse 3-year survival, regional recurrence, or distant recurrence rates compared with those treated with axillary lymph node dissection. To extend the recommendation of axillary lymph node dissection omission even in patients treated with mastectomy, a sub-analysis of the SINODAR-ONE trial is presented here.

**Methods:**

Patients with T1–2 breast cancer and no more than two metastatic sentinel lymph nodes undergoing mastectomy were analysed. After sentinel lymph node biopsy, patients were randomly assigned to receive either axillary lymph node dissection followed by adjuvant treatment (standard arm) or adjuvant treatment alone (experimental arm). The primary endpoint was overall survival. The secondary endpoint was recurrence-free survival.

**Results:**

A total of 218 patients were treated with mastectomy; 111 were randomly assigned to the axillary lymph node dissection group and 107 to the sentinel lymph node biopsy-only group. At a median follow-up of 33.0 months, there were three deaths (two deaths in the axillary lymph node dissection group and one death in the sentinel lymph node biopsy-only group). There were five recurrences in each treatment arm. No axillary lymph node recurrence was observed. The 5-year overall survival rates were 97.8 and 98.7 per cent in the axillary lymph node dissection treatment arm and the sentinel lymph node biopsy-only treatment arm, respectively (*P* = 0.597). The 5-year recurrence-free survival rates were 95.7 and 94.1 per cent in the axillary lymph node dissection treatment arm and the sentinel lymph node biopsy treatment arm, respectively (*P* = 0.821).

**Conclusion:**

In patients with T1–2 breast cancer and one to two macrometastatic sentinel lymph nodes treated with mastectomy, the overall survival and recurrence-free survival rates of patients treated with sentinel lymph node biopsy only were not inferior to those treated with axillary lymph node dissection. To strengthen the conclusion of the trial, the enrolment of patients treated with mastectomy was reopened as a single-arm experimental study.

**Registration number:**

NCT05160324 (http://www.clinicaltrials.gov)

## Teaser

In patients with cN0 T1–2 breast cancer and one to two macrometastatic sentinel lymph nodes treated with mastectomy, the overall survival and recurrence-free survival rates of patients treated with sentinel lymph node biopsy only were not inferior to those treated with axillary lymph node dissection. To strengthen the conclusion of the trial, the enrolment of breast cancer patients with one to two positive sentinel lymph nodes treated with mastectomy was reopened. The long-term oncological outcomes in terms of survival and recurrence and the prognostic impact of the omission of axillary lymph node dissection in these patients remain to be seen.

## Introduction

Axillary lymph node dissection (ALND) has always been part of breast cancer (BC) treatment^1^. Two large RCTs demonstrated no survival or recurrence advantage of ALND over less extensive surgery in BC patients with clinically negative nodes (cN0)^2,3^. Despite this, ALND remained the routine operation for axillary staging and locoregional control in BC treatment, conferring a high morbidity burden, due to frequent lymphoedema, pain, nerve damage, and decreased range of motion. In the 1990s, sentinel lymph node (SLN) biopsy (SLNB) was introduced into the BC surgical treatment algorithm^4^. In prospective RCTs, this simple and accurate axillary staging procedure was compared with ALND. Equivalent regional control, recurrence-free survival (RFS), and overall survival (OS) was shown if the SLN was negative^5–8^.

Efforts to limit redundant axillary surgical management have been ongoing over the last decade. The American College of Surgeons Oncology Group (ACOSOG) Z0011 trial randomized 891 women with cN0 BC and up to two positive SLNs detected after breast-conserving surgery (BCS) to either ALND or observation^9^. No statistically significant difference in OS was found after 9.3 years of follow-up^10^. The SINODAR-ONE RCT was designed to overcome limitations of the Z0011 study by enrolling BC patients undergoing both BCS and mastectomy^11^. The initial study results showed similar outcomes (3-year survival, regional recurrence, and distant recurrence rates) in BC patients with T1–2 tumours and one to two SLN macrometastases treated with BCS and SLNB only when compared with those treated with ALND^12^. According to the American Society of Clinical Oncology (ASCO) 2017 guidelines, there is insufficient evidence to recommend no further axillary surgery beyond SLNB for women with early BC and one to two SLN metastases selected for mastectomy^13,14^. Management of the axilla in patients with cN0 T1–2 BC with positive SLNB operated on with mastectomy is highly debated. Recent retrospective studies have demonstrated that ALND does not improve post-mastectomy survival outcomes among patients with a positive SLNB^15–17^. The use of ALND in these patients has, moreover, steadily decreased in routine clinical practice^18^. To extend the recommendation of ALND omission even in patients treated with mastectomy, a sub-analysis of the SINODAR-ONE trial is presented here.

## Methods

### Patient characteristics, study design, and endpoints

The SINODAR-ONE multicentre RCT was registered at ClinicalTrials.gov (ID NCT05160324) and approved by the institutional review boards of all participating centres. All patients provided written informed consent for treatment and clinical data acquisition. Patients with histologically confirmed invasive BC with a tumour size less than or equal to 5 cm, cN0, and no more than two metastatic SLNs were eligible for participation. In the present analysis, only patients operated on with mastectomy were included. The study design and endpoints have been described elsewhere^11,12^. Briefly, after SLNB, patients were randomly assigned (1 : 1 ratio) to receive either ALND followed by adjuvant treatment (standard arm) or adjuvant treatment alone with no further axillary surgery (experimental arm). The primary and secondary endpoints were OS and RFS respectively. Adjuvant therapy included chemotherapy, radiotherapy (RT), endocrine treatment, and/or Human Epidermal Growth Factor Receptor 2 (HER2)-targeted treatment, as appropriate. Patients were assessed for disease recurrence with physical examination every 6 months for the first 5 years, then yearly. This included annual mammography and ultrasonography of breast and axilla.

### Statistical analysis

Patients were selected from the same database managed by the Clinical Trials Centre of the IRCCS Ospedale Policlinico San Martino (Genoa, Italy), with the same observation interval. Initially, 2000 patients were planned for enrolment with a minimum 5-year follow-up. The first patient was enrolled in April 2015. Patients were enrolled from 52 Italian institutions. Trial enrolment was ceased prematurely in April 2020 due to low accrual rates and fewer events than expected. The primary endpoint was OS, defined as the interval between the randomization date and the date of last contact or death from any cause. The secondary endpoint was RFS, defined as regional RFS (no axillary recurrence) or distant RFS. The alternative hypothesis was that patients with T1–2 BC presenting one to two macrometastatic SLNs treated with SLNB only did not present higher mortality and recurrence rates compared with those treated with ALND. For this reason, the standard and experimental arms were compared in terms of OS and RFS using the Kaplan–Meier analysis method and the log rank test. Both intention-to-treat (ITT) and per-protocol (PP) populations were analysed. The 5-year cumulative incidence of mortality and recurrence and 95 per cent confidence intervals of the incidence rate per 100 patients were calculated. The cut-off for statistical significance was set at *P* < 0.050. Statistical analyses were performed using SPSS (IBM, Armonk, NY, USA; 25.0).

## Results

### Patient and tumour characteristics

**Fig. 1:**
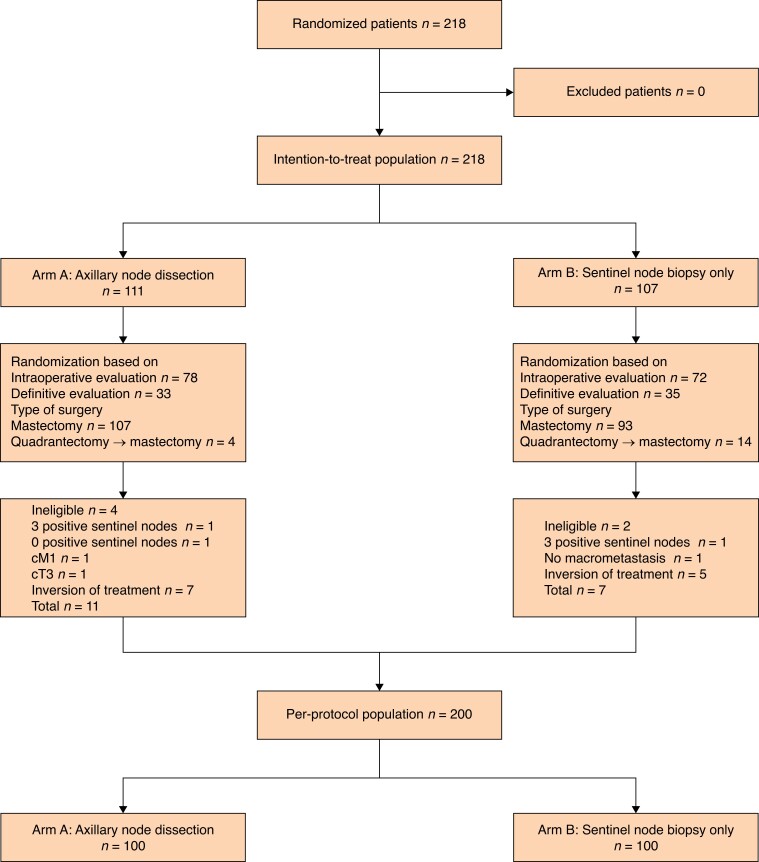
CONSORT flow diagram Mastectomy-group sub-analysis Consolidated Standards of Reporting Trials (CONSORT) flow diagram showing the phases of randomization and selection of 218 patients with T1–2 breast cancer and one to two SLN macrometastases undergoing mastectomy and axillary lymph node dissection or sentinel lymph node biopsy only.

Overall, 218 patients were treated with mastectomy, with 111 randomly assigned to the ALND group and 107 to the SLNB-only group (ITT population; *Fig. 1*). The majority of patients (150 patients) were randomized based on an intraoperative SLNB assessment. After excluding 18 patients for change of axillary treatment (12 patients) or ineligibility (6 patients), a more conservative analysis was performed on the PP population (200 patients) (*Fig. 1*). The mean(s.d.) age of the patients was 53.4(9.4) years and 107 (49.1 per cent) patients were premenopausal. Eighteen (8 per cent) patients underwent mastectomy after prior BCS due to positive resection margins. The mean(s.d.) tumour size was 24.7(12.7) mm and the cancers were unifocal in 118 (54.1 per cent) patients. The most common molecular subtype (118 patients (54.1 per cent)) was luminal B-like BC. Patient and tumour characteristics are summarized in *Table 1*. The CONSORT checklist is available in Supplementary material.

**Table 1:** Clinical characteristics by treatment arm

	ALND (*n* = 111)	SLNB only (*n* = 107)	All (*n* = 218)
**Clinical characteristics**			
Age (years), mean(s.d.)	53.8 (9.1)	53.1 (9.7)	53.4 (9.4)
Menopausal status			
Premenopausal	54 (48.7)	53 (49.5)	107 (49.1)
Perimenopausal	2 (1.8)	5 (4.7)	7 (3.2)
Postmenopausal	55 (49.6)	49 (45.8)	104 (47.7)
Breast surgery			
Mastectomy	107 (96.4)	93 (86.9)	200 (91.7)
BCS → mastectomy	4 (3.6)	14 (13.1)	18 (8.3)
**Histopathological characteristics**			
Tumour type			
Unifocal	59 (53.2)	59 (55.1)	118 (54.1)
Multifocal	26 (23.4)	27 (25.2)	53 (24.3)
Multicentric	26 (23.4)	21 (19.6)	47 (21.6)
Tumour size (mm), mean(s.d.)	26.3 (13.3)	23.1 (12.0)	24.7 (12.7)
pT stage			
pT1a	1 (0.9)	1 (0.9)	2 (0.9)
pT1b	2 (1.8)	9 (8.4)	11 (5.1)
pT1c	44 (39.6)	43 (40.2)	87 (39.8)
pT2	56 (50.5)	51 (47.7)	107 (49.1)
pT3	8 (7.2)	3 (2.8)	11 (5.1)
Histological subtype			
Invasive ductal carcinoma NST	67 (60.4)	74 (69.2)	141 (64.7)
Invasive lobular carcinoma	33 (29.7)	26 (24.3)	59 (27.1)
Tubular carcinoma	1 (0.9)	0 (0)	1 (0.5)
Mucinous carcinoma	1 (0.9)	1 (0.9)	2 (0.9)
Apocrine carcinoma	1 (0.9)	1 (0.9)	2 (0.9)
Invasive papillary carcinoma	2 (1.8)	1 (0.9)	3 (1.4)
Mixed ductal-lobular carcinoma	5 (4.5)	1 (0.9)	6 (2.8)
Other	1 (0.9)	3 (2.8)	4 (1.8)
Grade			
G1	9 (8.1)	12 (11.2)	21 (9.6)
G2	68 (61.3)	67 (62.6)	135 (61.9)
G3	33 (29.7)	28 (26.2)	61 (28.0)
GX	1(0.9)	0 (0)	1 (0.5)
Lymphatic invasion			
No	70 (63.1)	67 (62.6)	137 (62.8)
Yes	41 (36.9)	40 (37.4)	81 (37.2)
Vascular invasion			
No	66 (59.5)	67 (62.6)	133 (61.0)
Yes	45 (40.5)	40 (37.4)	85 (39.0)
Skin involvement			
No	104 (93.7)	97 (90.7)	201 (92.2)
Yes	7 (6.3)	10 (9.3)	17 (7.8)
Intraductal component			
≤25%	83 (74.8)	76 (71.0)	159 (72.9)
>25%	28 (25.2)	31 (29.0)	59 (27.1)
Hormone receptor status			
OR− PGR−	8 (7.2)	6 (5.6)	14 (6.4)
OR+ PGR−	9 (8.1)	6 (5.6)	15 (6.9)
OR− PGR+	0 (0)	0 (0)	0 (0)
OR+ PGR+	94 (84.7)	94 (87.9)	188 (86.2)
Missing value	0 (0)	1 (0.9)	1 (0.5)
Ki67			
0–13%	32 (28.8)	38 (35.5)	70 (32.1)
>14%	79 (71.2)	69 (64.5)	148 (67.9)
HER2 status			
Negative	92 (82.9)	96 (89.7)	188 (86.2)
Positive	15 (13.5)	9 (8.4)	24 (11.0)
Not evaluable	4 (3.6)	1 (0.9)	5 (2.3)
Missing value	0 (0)	1 (0.9)	1 (0.4)
Molecular subtype*			
Luminal A-like	29 (26.1)	36 (33.6)	65 (29.8)
Luminal B-like	60 (54.1)	58 (54.2)	118 (54.1)
HER2+	15 (13.5)	9 (8.4)	24 (11.0)
Triple negative	3 (2.7)	2 (1.9)	5 (2.3)
Missing value	4 (3.6)	2 (1.9)	6 (2.8)

Values are *n* (%) unless otherwise indicated. *Molecular subtype according to the St Gallen 2013 classification.^19^ HER2 evaluated either by immunohistochemistry or *in situ* hybridization, according to the American Society of Clinical Oncology College of American Pathologists guidelines. ALND, axillary lymph node dissection; SLNB, sentinel lymph node biopsy; BCS, breast-conserving surgery; NST, no special type; OR, oestrogen receptor; PGR, progesterone receptor; HER2, Human Epidermal Growth Factor Receptor 2.

### Treatment results

At randomization, the two different arms of axillary treatment presented a comparable SLN status, with a median number of 2 (interquartile range (i.q.r.) 1–3) removed SLNs and a median number of 1 (i.q.r. 1–1) positive SLN in each group. Overall, one patient in the ITT population had SNL micrometastasis and was treated with SLNB only. Additionally, one patient in the ITT population was incorrectly treated with ALND because no macrometastasis was found in the SLN at final pathology.

In the ALND treatment group, the median number of non-SLNs identified at final pathology was 15 (i.q.r. 11–21) and 53 of 111 (47.8 per cent) patients had additional metastases in lymph nodes removed by axillary dissection. The median number of metastatic non-SLNs was 1 (i.q.r. 0–2). Axillary lymph node data are summarized in *Table 2*.

**Table 2:** Axillary lymph node data by treatment arm

	ALND (*n* = 111)	SLNB only (*n* = 107)	All (*n* = 218)
**SLN outcome at randomization**			
Number of SLNs, median (i.q.r.)	2 (1–3)	2 (1–3)	2 (1–3)
Number of positive SLNs, median (i.q.r.)	1 (1–1)	1 (1–1)	1 (1–1)
**SLN at final pathology**			
No metastases	1 (0.9)	0 (0)	1 (0.5)
Micrometastases only	0 (0)	1 (0.9)	1 (0.5)
Macrometastases only	99 (89.2)	94 (87.9)	193 (88.5)
Micrometastases and macrometastases	11 (9.9)	12 (11.2)	23 (10.6)
**Non-SLNs at final pathology**			
Number of non-SLNs, median (i.q.r.)	15 (11–21)	–	15 (11–21)
Number of positive non-SLNs, median (i.q.r.)	1 (0–2)	–	1 (0–2)
1 positive non-SLN	26 (23.4)	–	26 (11.9)
2 positive non-SLNs	9 (8.1)	–	9 (4.6)
3 positive non-SLNs	5 (4.5)	–	5 (2.3)
>3 positive non SLNs	13 (11.7)	–	13 (6.0)

Values are *n* (%) unless otherwise indicated. ALND, axillary lymph node dissection; SLNB, sentinel lymph node biopsy; SNL, sentinel lymph node; i.q.r., interquartile range; –, not applicable.

In terms of adjuvant therapy, 38 (17 per cent) patients received RT (30 (27 per cent) and 8 (8 per cent) patients in the ALND arm and the SLNB-only arm respectively). Chemotherapy was administered to 116 (53.2 per cent) patients (63 of 111 (56.8 per cent) patients in the ALND arm and 53 of 107 (49.5 per cent) patients in the SLNB-only arm). Paclitaxel and anthracycline combination chemotherapy was the most common administered type in both groups. Trastuzumab was administered to 20 (9 per cent) patients and ribociclib was administered to 1 (1 per cent) patient. Overall, 196 (89.9 per cent) patients were treated with adjuvant endocrine treatment and most patients (46.8 per cent) received an aromatase inhibitor.

### Overall survival

In the ITT population, there were three deaths (two BC-related deaths in the ALND arm and one death from a second primary malignancy in the SLNB-only arm) at a median follow-up of 33.0 (i.q.r. 19.1–45.4) months. The 5-year cumulative incidence of mortality was 5.8 and 6.1 per cent in the ALND arm and the SLNB-only arm respectively (*P* = 0.617; *Table 3*). In the PP population, there was one death in each treatment arm, with a 5-year cumulative incidence of mortality of 5.6 and 6.1 per cent in the ALND arm and the SLNB-only arm respectively (*P* = 0.949; *Table 3*).

**Table 3:** Comparison of event rates by treatment arm

Outcome	ALND	SLNB only	*P*
**ITT population (*n* = 111 for ALND and *n* = 107 for SLNB)**			
Mortality			
No. of events	2	1	
5-year cumulative incidence, %	5.8	6.1	
Incidence rate per 100 patients (95% c.i.)	0.7 (0.1,2.4)	0.4 (0.0,2.0)	
Rate ratio (95% c.i.)	1 (reference)	0.6 (0.03,5.75)	0.617
Recurrences			
No. of events	5	5	
5-year cumulative incidence, %	2.9	3.3	
Incidence rate per 100 patients (95% c.i.)	0.9 (0.5,1.6)	1.2 (0.7,2.1)	
Rate ratio (95% c.i.)	1 (reference)	1.1 (0.31,3.95)	0.882
**PP population (*n* = 100 for ALND and *n* = 100 for SLNB)**			
Mortality			
No. of events	1	1	
5-year cumulative incidence, %	5.6	6.1	
Incidence rate per 100 patients (95% c.i.)	0.4 (0.0,2.0)	0.4 (0.0,2.2)	
Rate ratio (95% c.i.)	1 (reference)	1.1(0.04,27.63)	0.949
Recurrences			
No. of events	4	5	
5-year cumulative incidence, %	2.9	3.3	
Incidence rate per 100 patients (95% c.i.)	1.5 (0.4,3.7)	2.0 (0.6,4.6)	
Rate ratio (95% c.i.)	1 (reference)	1.4 (0.36,5.51)	0.643

ALND, axillary lymph node dissection; SLNB, sentinel lymph node biopsy; ITT, intention-to-treat; PP, per-protocol.

**Fig. 2:**
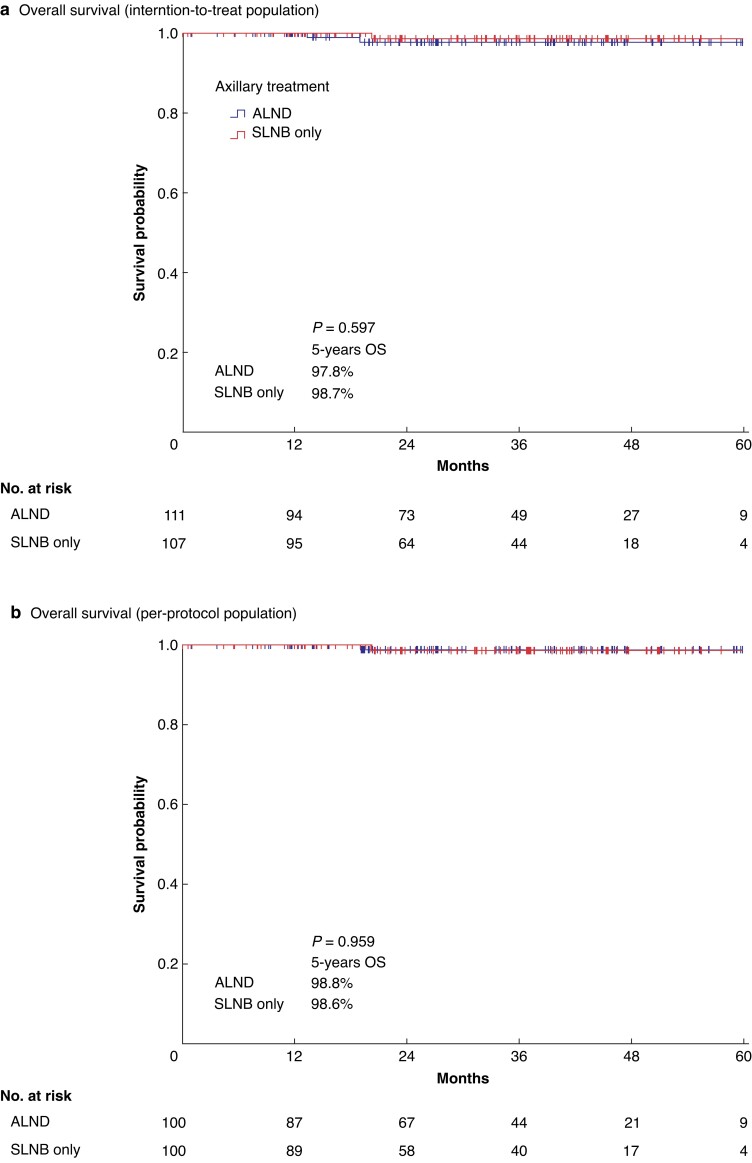
Overall survival curves **a** Overall survival curves for the intention-to-treat population of patients with T1–2 breast cancer and one to two sentinel lymph node macrometastases undergoing mastectomy and either standard axillary treatment (axillary lymph node dissection) or experimental treatment (sentinel lymph node biopsy only). **b** Overall survival curves for the per-protocol population of patients with T1–2 breast cancer and one to two sentinel lymph node macrometastases undergoing mastectomy and either standard axillary treatment (axillary lymph node dissection) or experimental treatment (sentinel lymph node biopsy only). OS, overall survival.

In cN0 T1–2 BC patients undergoing mastectomy with one to two positive SLNs, the omission of ALND did not yield statistically inferior survival results for either the ITT population or the PP population (*Fig. 2*). In the ITT population, the 5-year OS rates were 97.8 and 98.7 per cent, in the ALND arm and the SLNB-only arm respectively (*P* = 0.597). In the PP population, the 5-year OS rates were 98.8 and 98.6 per cent, in the ALND arm and the SLNB-only arm respectively (*P* = 0.959).

### Recurrence-free survival

In the ITT population, there were five recurrences in each treatment arm, with a 5-year cumulative incidence of recurrence of 2.9 and 3.3 per cent in the ALND arm and the SLNB-only arm respectively (*P* = 0.882; *Table 3*). Bone metastasis was the most common site of distant recurrence (five instances), followed by liver metastasis (three instances) and an ipsilateral local recurrence (two instances). No axillary lymph node recurrences were observed.

In the PP population, there were nine recurrences (four in the ALND arm and five in the SLNB-only arm), with a 5-year cumulative incidence of recurrence of 2.9 and 3.3 per cent in the ALND arm and the SLNB-only arm respectively (*P* = 0.643; *Table 3*).

**Fig. 3:**
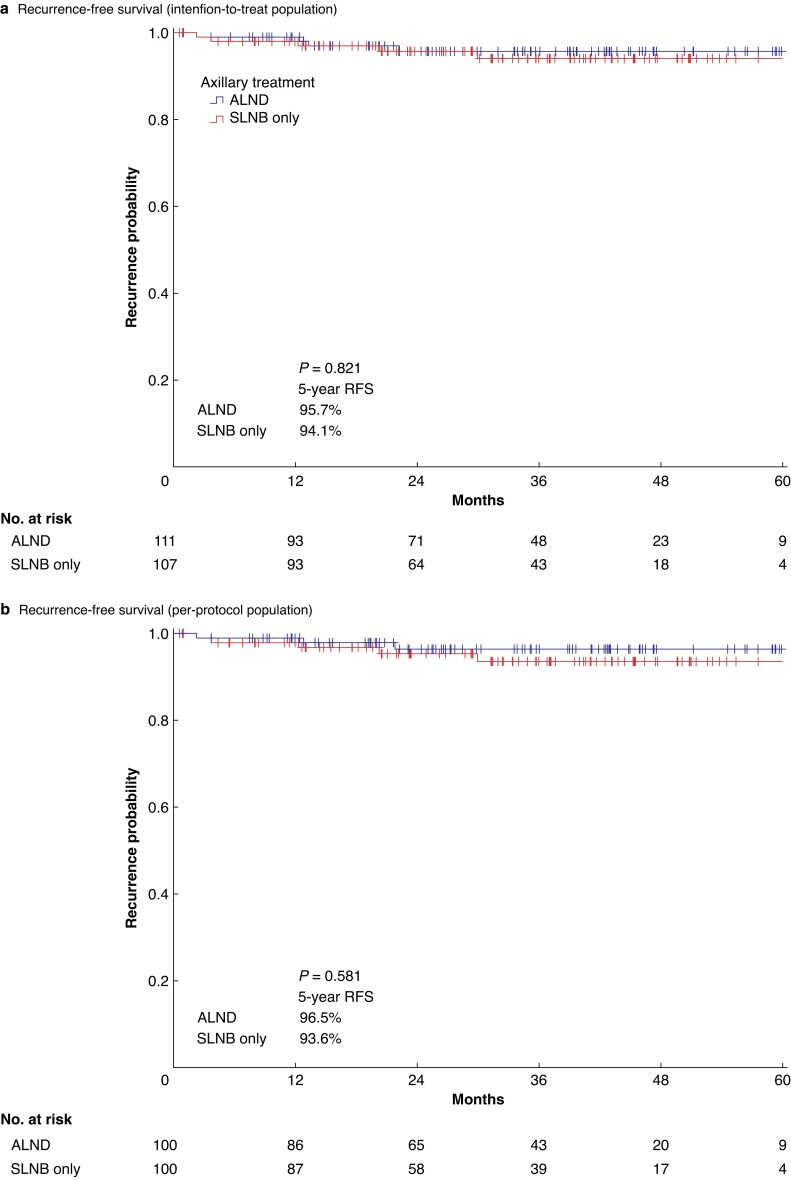
Recurrence-free survival curves **a** Recurrence-free survival curves for the intention-to-treat population of patients with T1–2 breast cancer and one to two sentinel lymph node macrometastases undergoing mastectomy and either standard axillary treatment (axillary lymph node dissection) or experimental treatment (sentinel lymph node biopsy only). **b** Recurrence-free survival curves for the per-protocol population of patients with T1–2 breast cancer and one to two sentinel lymph node macrometastases undergoing mastectomy and either standard axillary treatment (axillary lymph node dissection) or experimental treatment (sentinel lymph node biopsy only). RFS, recurrence-free survival.

Preserving axillary lymph nodes in cN0 T1–2 BC patients undergoing mastectomy with one to two positive SLNs by omitting ALND did not lead to increased recurrence rates when compared with patients treated with ALND (*Fig. 3*). In the ITT population, the 5-year RFS rates were 95.7 and 94.1 per cent in the ALND arm and the SLNB-only arm respectively (*P* = 0.821). In the PP population, the 5-year RFS rates were 96.5 and 93.6 per cent in the standard arm and the experimental treatment arm respectively (*P* = 0.581).

## Discussion

Over the past two decades, the management of early BC has focused on less radical surgeries and axillary management has undergone a paradigm shift along this path. The results of the SINODAR-ONE and ACOSOG Z0011 clinical trials have suggested that ALND can be avoided in cN0 patients with up to two macroscopically positive SLNs who undergo BCS. For patients treated with mastectomy, the clinical value of ALND has not been well defined. There is still controversy over the de-escalation of axillary surgery in patients receiving mastectomy with one to two macrometastatic SLNs, and it remains unclear whether these results can be safely applied to these patients^20,21^.

Retrospective analyses have, however, evaluated the outcomes of BC patients selected for mastectomy with omission of ALND, despite one to two SLN macrometastases^15,17,22–27^. Gao *et al*.^22^ performed a large multi-institutional study on 1161 BC patients with one to two positive SLNs. Overall, 763 patients underwent mastectomy, of which 84 received SLNB only. There were no differences in adjusted RFS and OS between the SLNB-only and ALND groups, thus indicating that omitting ALND has no impact on oncological outcomes. To note, 26 of 84 (31.0 per cent) patients treated with mastectomy and SLNB merely had micrometastases in the SLNs. Milgrom *et al*.^23^ analysed the outcome of 535 patients with early-stage BC and positive SLNs, treated with breast surgery (210 treated with mastectomy and 325 treated with BCS), who did not receive ALND. Patients treated with mastectomy had favourable oncological outcomes even in the absence of ALND. Axillary failure rates did not differ significantly from patients selected for BCS. The 4-year local and regional failure rates were 1.7 and 1.2 per cent among patients treated with mastectomy and 1.4 and 1.0 per cent among patients treated with BCS respectively. FitzSullivan *et al*.^17^ evaluated 525 patients with BC and positive SLNs treated with mastectomy, including 58 patients who did not undergo ALND and/or axillary RT. At a median follow-up of 66 months, the incidence of axillary recurrences was not statistically different between patients who did not receive further axillary treatment and those who underwent ALND or axillary RT (10-year rate 3.8 *versus* 1.6 *versus* 0 per cent respectively).

The available data on axillary management in cN0 T1–2 BC patients undergoing mastectomy with one to two positive SLNs is thus limited to a small number of patients in retrospective studies. To overcome these limitations and evaluate the oncological results of ALND omission, some clinical trials have included cN0 BC patients with positive SLNs selected for mastectomy in their protocols. The BOOG 2013-07 trial^28^ is a non-inferiority, randomized, controlled multicentre study that aims to clarify whether patients with cN0 T1–2 BC undergoing mastectomy benefit from ALND or may undergo SLNB only, if three or fewer lymph nodes contain micrometastases or macrometastases. In this trial, completion of axillary treatment may consist of ALND or axillary RT. The hypothesis is that completion axillary treatment can be safely omitted in BC patients with positive SLNs undergoing mastectomy. The primary endpoint is the regional recurrence rate at 5 years and secondary endpoints include the number of delayed axillary treatments, distant disease-free survival, OS, the local recurrence rate, the other-regional recurrence rate, the contralateral BC rate, the percentage difference in the administration of postoperative RT, the axillary morbidity rate, and quality of life. The ongoing POSNOC trial^29^ is a non-inferiority, multicentre study that includes women with BC with one to two macrometastases. This trial specifically addresses the question of ALND omission in both women treated with BCS and mastectomy and whether adjuvant therapy alone is non-inferior to adjuvant therapy plus axillary treatment, in terms of 5-year axillary recurrence. The POSNOC trial will thus try to provide a more definitive answer regarding the safety of SLNB only in women with one to two SLNs with macrometastases treated with BCS or mastectomy. The SENOMAC trial^30^ is a non-inferiority, international study randomizing cN0 T1–3 BC patients with one to two positive SLNs to either ALND or no further axillary surgery. The primary endpoint is OS at 5 years. Secondary endpoints include BC recurrence, disease-free and BC-specific survival, and contralateral BC, as well as arm morbidity and quality of life measured using questionnaires at 1, 3, and 5 years. Adjuvant systemic therapies are administered in accordance with the national clinical guidelines for each participating country. Breast RT is given to all patients undergoing BCS and post-mastectomy RT is given to all patients except for those with T1–2 and G1 tumours with only one SLN macrometastasis. The first published outcomes are related to one of the secondary outcomes and show that, 1 year after surgery, arm morbidity is significantly worse in the ALND arm^31^. The European Organisation for Research and Treatment of Cancer 10981-22023 AMAROS trial^32^ investigated whether axillary RT could replace ALND in patients with cN0 T1–2 BC and a positive SLN. The planned non-inferiority test was underpowered because of the low number of events; however, after 10-year follow-up, both the axillary RT and ALND groups showed very low axillary recurrence (0.9 *versus* 1.6 per cent in the ALND group and the axillary RT group respectively) and similar OS rates (14.0 *versus* 16.4 per cent in the ALND group and the axillary RT group respectively)^33^. Significantly lower lymphoedema rates were observed in the axillary RT group and the need for any axillary therapy in patients with limited nodal disease was questioned^33^. These results and trials underline the importance of ongoing attempts to safely de-escalate axillary surgery. It should be noted, however, that the rate of axillary tumour left behind when omitting ALND was 47.8 per cent in the present study, as assessed by findings at ALND in the standard treatment arm. Nevertheless, these trials provide evidence that the combination of RT and systemic treatments may sufficiently control and treat axillary disease^34^.

The present sub-analysis of the SINODAR-ONE trial evaluated 218 patients with cN0 T1–2 BC and one to two SLN macrometastases treated with mastectomy. Out of these, 107 patients were assigned to the experimental treatment arm and received SLNB only. Similar to the authors’ previous results^12^, the survival and recurrence rates of patients treated with SLNB only were not inferior to those treated with ALND. Given the low number of patients treated with mastectomy, however, there is no certainty that ALND omission can also be extended to this subgroup. To collect further evidence regarding the safety of SLNB only in patients selected for mastectomy, the reopening of the SINODAR-ONE trial for enrolment of such patients, as part of a single-arm experimental study, started in June 2022. The aim being to enrol 400 patients treated with mastectomy.

The present study has limitations. A subgroup analysis could have led to false-negative results because of the two treatment groups being much smaller than the original study population. The statistical power could, moreover, have been influenced by poor accrual rates and fewer than anticipated events. Additionally, even if post-mastectomy RT rates were significantly different between the treatment arms, no additional or more detailed adjuvant RT data are currently available. Finally, the present trial has a relatively short follow-up time. To strengthen the conclusion of the trial, enrolment of BC patients with one to two positive SLNs and selected for mastectomy was reopened.

## Collaborators

**SINODAR-ONE Collaborative Group**

Massimo Maria Grassi (Breast Unit, Humanitas Gavazzeni Clinical Institute, Bergamo, Italy); Olindo Custodero (UOSVD Chirurgia Senologica – Breast Unit ASL BA P.O. San Paolo, Bari, Italy); Vito Leopoldo Troilo (UOSVD Chirurgia Senologica – Breast Unit ASL BA P.O. San Paolo, Bari, Italy); Mario Taffurelli (IRCCS – Azienda Ospedaliero Universitaria di Bologna – Policlinico di Sant’Orsola, Bologna, Italy); Maria Cristina Cucchi (U.O. Chirurgia Senologica Dipartimento Chirurgie Specialistiche USL di Bologna, Italy); Valentina Galluzzo (U.O. Chirurgia Senologica Dipartimento Chirurgie Specialistiche USL di Bologna, Italy); Carlo Cabula (Oncologia e Senologia Azienda di Rilievo Nazionale Alta Specializzazione ARNAS – Cagliari, Italy); Roberta Cabula (Oncologia e Senologia Azienda di Rilievo Nazionale Alta Specializzazione ARNAS – Cagliari, Italy); Maria Grazia Lazzaretti (Unità semplice di Chirurgia Senologica, Ospedale Ramazzini di Carpi, AUSL Modena, Italy); Francesco Caruso (Humanitas Istituto Clinico Catanese – Contrada Cubba SP 54 n. 11, 95045, Misterbianco (CT), Italy); Gaetano Castiglione (Humanitas Istituto Clinico Catanese – Contrada Cubba SP 54 n. 11, 95045, Misterbianco (CT), Italy); Simona Grossi (U.O.C. di Chirurgia Generale a indirizzo Senologico – EUSOMA Breast Centre ASL 2 Abruzzo, Italy); Maria Saveria Tavoletta (U.O.C. di Chirurgia Generale a indirizzo Senologico – EUSOMA Breast Centre ASL 2 Abruzzo, Italy); Camilla Rossi (U.O. Chirurgia Senologica, Ospedale degli Infermi, Viale Stradone 9, Faenza, Italy); Annalisa Curcio (Chirurgia Senologica Ospedale di Forlì – Ravenna, Azienda USL della Romagna, Italy); Daniele Friedman (UO Clinica di Chirurgia Policlinico San Martino – IRCCS, Genoa, Italy); Piero Fregatti (UO Clinica di Chirurgia Policlinico San Martino – IRCCS, Genoa, Italy); Carla Magni (SSD Breast Unit ASST Lecco, Italy); Giovanni Tazzioli (Chirurgia Oncologica Senologica, Azienda Ospedaliera-Universitaria di Modena, Italy); Simona Papi (Chirurgia Oncologica Senologica, Azienda Ospedaliera-Universitaria di Modena, Italy); Riccardo Giovanazzi (Breast Unit – Surgery – San Gerardo Hospital, Monza, Italy); Camelia Chifu (Breast Unit – Surgery – San Gerardo Hospital, Monza, Italy); Rossella Bettini (UOC Chirurgia Senologica IRCCS Sacro Cuore Don Calabria, Presidio Ospedaliero Accreditato – Regione Veneto, Italy); Modestino Pezzella (UOC Chirurgia Senologica IRCCS Sacro Cuore Don Calabria, Presidio Ospedaliero Accreditato – Regione Veneto, Italy); Silvia Michieletto (Breast Surgery, Istituto Oncologico Veneto, Padova, Italy); Tania Saibene (Breast Surgery, Istituto Oncologico Veneto, Padova, Italy); Manuela Roncella (Breast Surgery Unit, Azienda Ospedaliero-Universitaria Pisana, Pisa, Italy); Matteo Ghilli (Breast Surgery Unit, Azienda Ospedaliero-Universitaria Pisana, Pisa, Italy); Andrea Sibilio (Oncology Department AUSL Romagna, Ravenna Hospital, Ravenna, Italy); Anna Cariello (Oncology Department AUSL Romagna, Ravenna Hospital, Ravenna, Italy); Saverio Coiro (Breast Surgery Unit, Azienda USL – IRCCS di Reggio Emilia, Italy); Giuseppe Falco (Breast Surgery Unit, Azienda USL – IRCCS di Reggio Emilia, Italy); Emanuele Zarba Meli (UOC Senologia, Azienda ospedaliera San Giovanni-Addolorata, Rome, Italy); Lucio Fortunato (UOC Senologia, Azienda ospedaliera San Giovanni-Addolorata, Rome, Italy); Luigi Ciuffreda (Chirurgia Senologica, Dipartimento Scienze Chirurgiche, IRCCS ‘Casa Sollievo della Sofferenza’ Opera di San Pio da Pietralcina, 71013, San Giovanni Rotondo (FG), Italy); Roberto Murgo (Chirurgia Senologica, Dipartimento Scienze Chirurgiche, IRCCS ‘Casa Sollievo della Sofferenza’ Opera di San Pio da Pietralcina, 71013, San Giovanni Rotondo (FG), Italy); Claudio Battaglia (Breast Unit, Civic Hospital of Sanremo, ASL 1 Imperiese, Sanremo, Italy); Luca Rubino (Breast Unit, Civic Hospital of Sanremo, ASL 1 Imperiese, Sanremo, Italy); Nicoletta Biglia (SCDU Gin e Ost, Ospedale Mauriziano Umberto primo, Torino, Italy); Valentina Bounous (SCDU Gin e Ost, Ospedale Mauriziano Umberto primo, Torino, Italy); Francesca Angela Rovera (Centro Ricerche in Senologia, Università degli Studi dell’Insubria, S.S.D. Breast Unit, ASST SetteLaghi, Varese, Italy); Corrado Chiappa (Centro Ricerche in Senologia, Università degli Studi dell’Insubria, S.S.D. Breast Unit, ASST SetteLaghi, Varese, Italy); Giovanni Pollini (UOC Chirurgia Senologica – Breast Unit, AOUI Verona, Italy); Sara Mirandola (UOC Chirurgia Senologica – Breast Unit, AOUI Verona, Italy); Graziano Meneghini (Breast Unit, Ospedale Montecchio Maggiore, Vicenza, Italy); Francesco Di Bartolo (Breast Unit, Ospedale Montecchio Maggiore, Vicenza, Italy); Oreste Davide Gentilini (Breast Surgery Unit, San Raffaele Hospital, Milan, Italy).

## Supplementary Material

znad215_Supplementary_Data

## Data Availability

The data that support the findings of this study are available as *Supplementary material*.
