# Supplementary figures and images for: Sentinel lymph node biopsy versus axillary lymph node dissection in breast cancer patients undergoing mastectomy with one to two metastatic sentinel lymph nodes: sub-analysis of the SINODAR-ONE multicentre randomized clinical trial and reopening of enrolment
Source: Br J Surg. 2023 Jul 20;110(9):1143–52. doi: 10.1093/bjs/znad215 (PMC10492188; doi:10.1093/bjs/znad215)

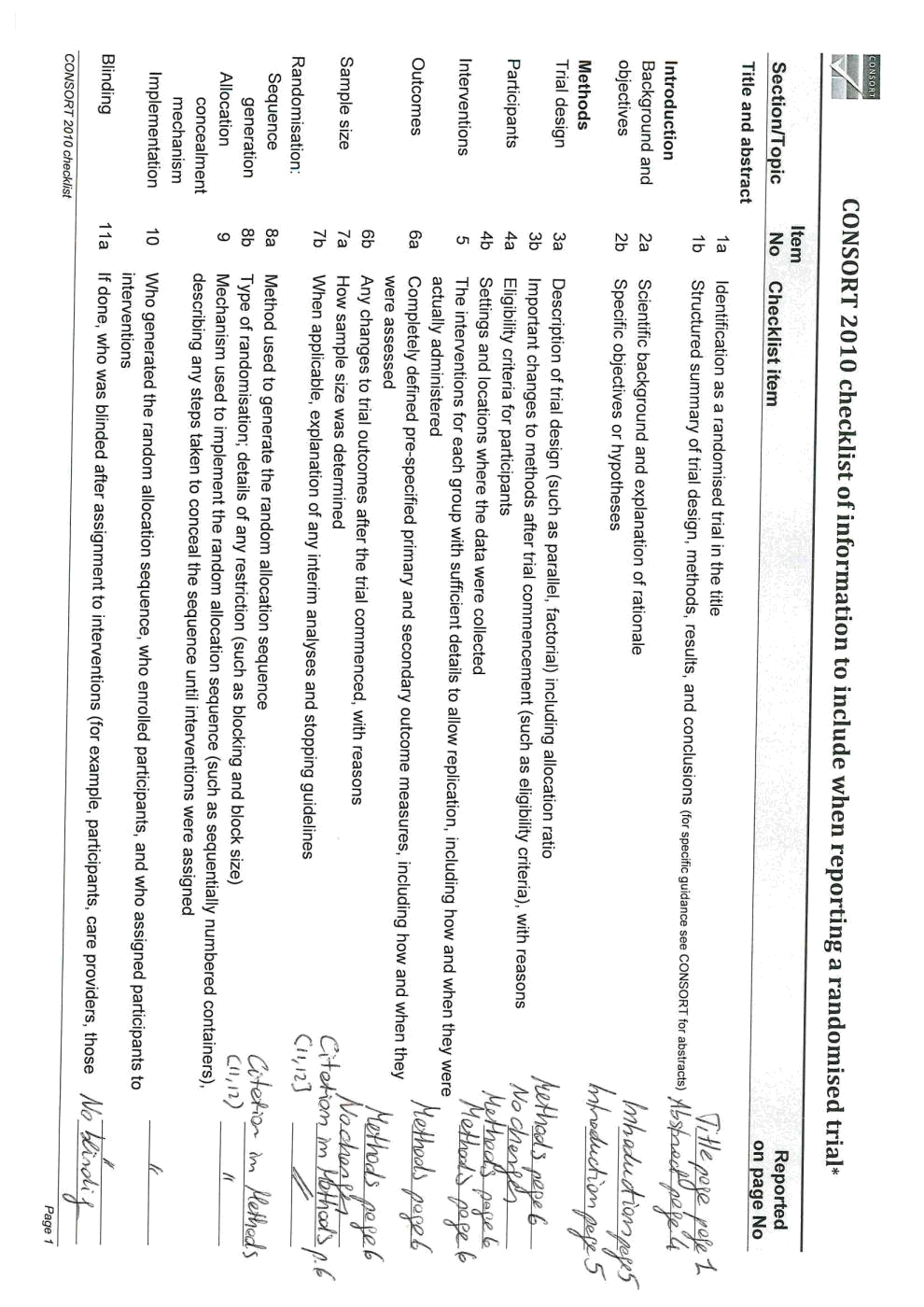

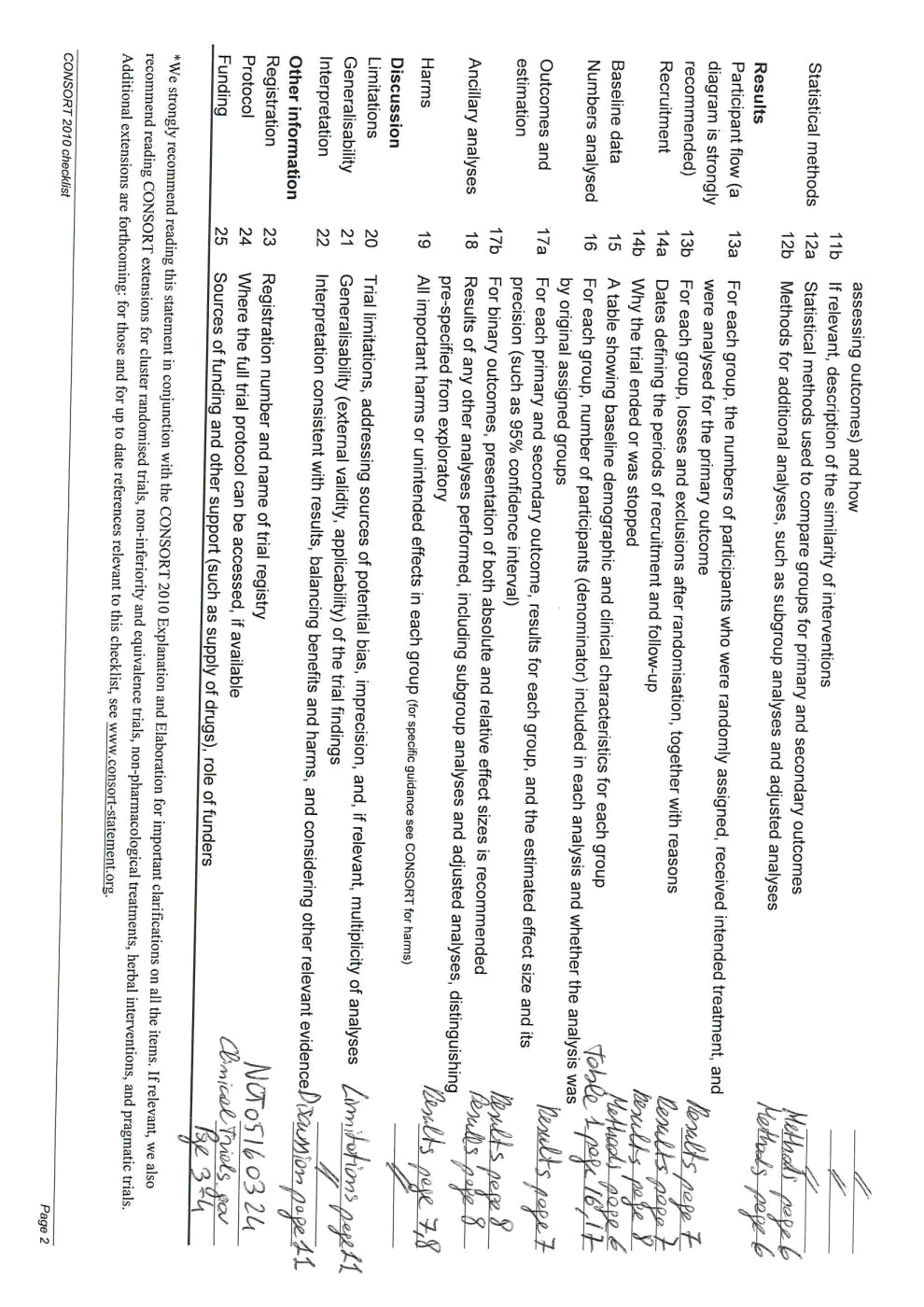

Supplement: znad215_Supplementary_Data [file znad215_supplementary_data.doc]
